# Effects of teriparatide on histomorphological features in a patient with an atypical femoral fracture and chronic kidney disease

**DOI:** 10.1097/j.pbj.0000000000000253

**Published:** 2024-06-19

**Authors:** Daniela Oliveira, Salomé Garcia, Luciano Pereira, Juliana Magalhães, Lúcia Costa, João Frazão, Carlos Vaz

**Affiliations:** a Rheumatology Department, Centro Hospitalar Universitário de São João, Porto, Portugal; b Center for Health Technology and Services Research (CINTESIS), Faculty of Medicine, University of Porto, Porto, Portugal; c Department of Medicine of Faculty of Medicine, University of Porto, Porto, Portugal; d Institute of Investigation and Innovation in Health, University of Porto, Porto, Portugal; e INEB—National Institute of Biomedical Engineering, University of Porto, Porto, Portugal; f USF BarcelSaúde do ACeS Cávado III Barcelos/Esposende, Barcelos, Portugal; g Nephrology Department, Centro Hospitalar Universitário de São João, Porto, Portugal

Atypical femoral fractures (AFFs) located at the femoral diaphysis are mainly described in patients undergoing prolonged treatment with drugs that suppress osteoclastic activity, namely bisphosphonates (BPs) and denosumab.^1,2^ Previous studies showed an increased risk of AFF in these patients, with incidence rates increasing from 1.8/100.000/year to 113/100.000/year with BP exposure from 2 to 9 years.^3,4^ The pathophysiology of AFF is not totally understood; however, it is thought that prolonged use of BP may lead to adynamic bone. Therefore, antiresorptive medications should be stopped immediately in these patients as the risk of an AFF in the contralateral femur exists. However, next pharmacological options remain to be clarified. Teriparatide (TPTD), a recombinant form of parathyroid hormone (PTH), is an antiosteoporotic agent with potent bone-forming effects that enhances bone healing in patients with delayed healing or nonunion.^5,6^ A number of studies have demonstrated beneficial radiographically and histologically effects of TPTD on fracture healing in various animal models.^7-9^ Nevertheless, the literature is scarce about the role of TPTD in the treatment of patients with a previous AFF, namely its benefit on histological findings and consequently its role in reducing the risk of fracture over time. In particular, there is not enough real-world data for health care professionals to adequately evaluate how safe and effective TPTD is for patients with a previous AFF and simultaneous chronic kidney disease (CKD). Thus, we report a case of a patient with an AFF and CKD treated with TPTD over 2 years with histological benefits.

We present a case of a 65-year-old man with a stage 3a CKD (creatinine of 1.4 mg/dL with an estimated glomerular filtration rate (GFR) by MDRD equation of 51 mL/min/1.73 m^2^) and osteoporosis with no personal history of fractures. This patient was admitted to the emergency department due to severe left trochanteric region pain following a low-impact fall. There were no smoking and alcoholic habits. No current or previous chronic steroid therapy, femoral neck fracture of his parents, and prolonged immobility in the past 6 months were reported. He was on alendronate (70 mg/weekly) and supplementation with calcium (1000 mg calcium/880 UI vitamin D3, one chewable tablet/day) and vitamin D (calcifediol 0.266 mg, 1 capsule/month) for the past 20 years. Pelvic X-ray revealed a fracture line in the femoral diaphysis, distal to the small trochanter, with orientation that was mainly transverse laterally and more oblique medially, forming a “spike” in the medial cortex. After an osteosynthesis procedure, the patient was referred to our Fracture Liaison Service.

**Figure 1.**
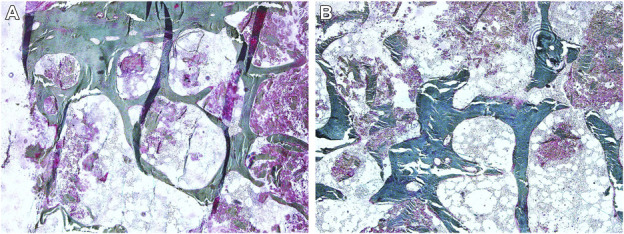
Tetracycline-labeled bone biopsy. Cortex. Staining modified Masson-Goldner trichome; original magnification× 50. First biopsy (A). Cortical thickness reduction. A zone of trabecular bone is observed, a reduction in the volume of trabecular bone, loss of the anastomosed pattern between the trabeculae, even at low magnification, a reduction in the osteoid surface is observed, and absence of bone cells. Second biopsy (B). A zone of trabecular bone is observed. In general, trabeculae preserved the anastomosed pattern. The reduction in bone volume is slight (improvement compared with the first biopsy). Even at low magnification, areas with the osteoid and erosion surface can be identified. For both, some artifacts resulting from the biopsy and staining procedure.

On investigation, creatinine was 1.3 mg/dL, with an estimated GFR by MDRD equation of 55.4 mL/min/1.73 m^2^ (stage 3a CKD). Ionized calcium, inorganic phosphate, and 25-OH-vitamin D were normal. Regarding serum bone turnover markers, alkaline phosphatase was 62 U/L (normal range 30–120), parathormone (PTH) was 26.7 pg/mL (normal range 10.0 and 65.0 pg/mL), osteocalcin was 18 ng/mL (normal range <41.3), and beta C‐terminal cross‐linking telopeptide of type I collagen (beta-crosslaps) was 0.17 ng/mL (normal range <0.32). Dorsal and lumbar spine radiographs revealed no vertebral fractures. Bone densitometry (GE-Lunar dual-energy X-ray film absorptiometry) revealed lumbar spine and femoral neck T-scores to be −2.9 and −1.9 (body mass density—BMD 0.672 g/cm^2^), respectively. For suspicion of adynamic bone disease, the patient performed a tetracycline-labeled bone biopsy. This biopsy showed absent osteoblastic surface and reduced osteoclastic, erosion, and osteoid surfaces (Fig. 1A). Fluorescent microscopy revealed few very weak tetracycline single labels. These findings were compatible with the diagnosis of low-turnover bone disease/adynamic bone disease. The main histomorphometry parameters are listed in Table 1.

**Table 1:** Histomorphometry parameters of two bone biopsies.

Histomorphometric parameters	Before teriparatide	After teriparatide	Reference range
BV/TV (%)	12.11	19.77	24.60 ± 7.10
OV/BV (%)	0.05	1.29	2.30 ± 2.40
OS/BS (%)	0.26	5.02	13.30 ± 11.10
ES/BS (%)	0.21	0.88	NS
OTh (µm)	4.64	8.23	11.2 ± 3.40
TbN (mm)	2.73	3.03	NS
Tb.sp (µm)	321.03	264.35	NS
ObS/BS (%)	0	0.46	1.60 ±3.30
OcS/BS (%)	0.10	0.12	0.03 ± 0.09
BFR/BS (µm^3^/µm^2^/year)*	NS	NS	0.061 ± 0.025
MS/BS (%)	1.14	2.21	NS
sL.S/BS	2.28	3.40	NS
MLT (d)	NS	NS	17.3 ± 6.5

* BFR/BS and MLT were not calculated due to the absence of tetracycline double labels. BV, bone volume; TV, tissue volume; OV, osteoid volume; OS, osteoid surface; BS, bone surface; OTh, osteoid thickness; TbN, trabecular number; Tb.sp, trabecular separation; ObS, osteoblast surface; OcS, osteoclast surface; MS, mineralizing surface; sL.S, single labeled surface; MLT, mineralization lag time; NS, not specified.

The patient maintained the supplementation of calcium and vitamin D and started TPTD (20 micrograms subcutaneous daily administration). After 2 years of TPTD, the patient repeated the bone biopsy showing an improvement of histological findings, namely in bone volume and bone formation parameters (Fig. 1B and Table 1). Currently, creatinine is 1.4 mg/dL, PTH 60.4 pg/mL, osteocalcin 23.4 ng/mL, and beta-crosslaps 0.26 ng/mL, with an improvement of 25.5% in femoral neck BMD (BMD 0.844 g/cm^2^ and T-score −1.7). During this follow-up period, no further fractures and no adverse events occurred.

We present a case of AFF characterized by histological findings confirming adynamic bone. While prolonged BP therapy over 20 years stands as a major risk factor, the presence of long-standing CKD also significantly contributed to this type of fracture. Despite the ideal duration of BP therapy not being clearly defined, therapy courses with these drugs exceeding 10 years are not currently recommended because they are associated with an increased risk of AFF.^10^ Nevertheless, approximately 20% of individuals with an AFF were not taking any bone antiresorptive treatment, and other conditions are thought to contribute such as CKD (adynamic bone).^11^ This patient was treated with TPTD over two years with an improvement of histomorphology findings, namely in bone volume and bone formation parameters with no fractures and no adverse events. Although healing BP-associated AFF is usually delayed because of suppressed bone turnover, TPTD appears to improve bone healing in patients with delayed healing or nonunion. Previous research involved patients with a BP-associated AFF and an average GFR of 78 mL/min/1.73 m^2^ treated with TPTD for one year showed an improvement in histomorphometry bone evaluation.^6^ Although this study included patients with stages 1–3 CKD, the authors did not discuss histomorphometry results specifically in these patients. Addressing fracture healing in patients with AFF is crucial, and it can be reached using anabolic therapy. However, the current evidence on the role of TPTD in reducing future fracture risk comes mainly from retrospective studies and small heterogeneous prospective studies.

Our case reinforces the importance of regular monitoring of fracture risk in patients on long-term antiosteoporotic treatment. Furthermore, this case highlights the role of TPTD in the treatment of AFF in a patient with CKD, improving histomorphology findings and reducing risk of future fracture. Randomized controlled trials with larger samples are needed to explore the role of TPTD in reducing future fracture risk in these patients.
